# Exploration of induced sputum BIRC3 levels and clinical implications in asthma

**DOI:** 10.1186/s12890-022-01887-2

**Published:** 2022-03-14

**Authors:** Lijuan Du, Changyi Xu, Zhimin Zeng, Fengjia Chen, Kun Tang, Yuxia Liang, Yubiao Guo

**Affiliations:** 1 Department of Pulmonary and Critical Care Medicine, The First Affiliated Hospital of Sun Yat-Sen University, No. 58 Zhongshan 2nd Road, Guangzhou, 510080 Guangdong China; 2 Institute of Respiratory Diseases of Sun Yat-Sen University, No. 58 Zhongshan 2nd Road, Guangzhou, 510080 Guangdong China

**Keywords:** Asthma, BIRC3, Inflammation, Induced sputum, R software

## Abstract

**Background:**

Baculoviral IAP repeat-containing 3 (BIRC3) which encodes a member of the IAP family of proteins upregulated in the asthma expression profile dataset. However, there was few research on studying the clinical implication of BIRC3 in asthma.

**Objective:**

To validate BIRC3 expression and its clinical implications in induced sputum of asthma.

**Methods:**

Based on the GSE76262 (118 asthma cases and 21 healthy controls) dataset, differentially expressed genes were screened using R software. Subsequently, BIRC3 mRNA and protein were clinically verified in induced sputum samples through quantitative real‐time polymerase chain reaction (qRT-PCR) and enzyme-linked immunosorbent assay (ELISA). Besides, the correlations between BIRC3 expression and asthmatic eosinophilic/allergic inflammation indicators (FeNO, IgE, and EOS%), pulmonary function (FEV_1_, FEV_1_% pred, FVC% pred, and FEV_1_/FVC), and inflammatory cytokines (IL-4, IL-5, IL-13, IL-25, IL-10, IL-33, and TSLP) were analyzed. Finally, BIRC3 mRNA was detected in human primary bronchial epithelial cells stimulated by cytokines (IL-4 or IL-13).

**Results:**

BIRC3 was screened as a candidate gene in the GSE76262, which was highly expressed in asthma. Highly expressed BIRC3 was positively correlated with eosinophilic and allergic indicators, including FeNO, blood eosinophil, and serum IgE. Moreover, BIRC3 protein was positively associated with inflammation cytokines, like IL-4, IL-5, IL-13, IL-25, IL-10, IL-33, and TSLP, while negatively correlated with FEV1, FEV1%pred, FVC% pred, and FEV1/FVC. Furthermore, the expression of BIRC3 could be induced in primary bronchial epithelial cells treated by cytokines IL-4 or IL-13.

**Conclusions:**

BIRC3 significantly increased in induced sputum of asthma and positively correlated with airway eosinophilic and peripheral blood allergic inflammation, type 2 cytokines, and airway obstruction. Increased BIRC3 might be involved in the pathogenesis of asthma by affecting the eosinophilic and allergic inflammation.

**Supplementary Information:**

The online version contains supplementary material available at 10.1186/s12890-022-01887-2.

## Background

Asthma, as one of the global common chronic airway inflammatory diseases, is characterized by recurrent wheezing, shortness of breath, chest tightness, and (or) cough, accompanied by reversible airflow limitation [1]. According to the global initiative for asthma (GINA) forecast, there will be about 400 million asthmatic patients in 2025, which imposes a heavy psychological and economic burden on society and the family [2].

Induced sputum is the most common established and noninvasive method to define the inflammatory phenotype of asthma, in which gene expression profiles could be used to identify diagnostic biomarkers and therapeutic targets [3–6]. Recently, with the rapid development of gene chip technology, large numbers of asthmatic sputum sequencing data have been uploaded to the public database GEO (Gene expression omnibus, https://www.ncbi.nlm.nih.gov/geo/) [7–9]. In our study, the GSE76262 dataset (Kuo et al.) [10] in induced sputum of asthma and healthy controls was downloaded for bioinformatic analysis, and the significantly upregulated BIRC3 gene was obtained.

In this study, we aimed to identify upregulated BIRC3 that may act as a sputum biomarker in asthma based on the microarray dataset of GSE76262, validate BIRC3 expression using qRT-PCR and ELISA in clinical patients’ samples, and explore the clinical implication of BIRC3 in induced sputum of asthma.

## Methods

### Bioinformatics analysis

The data of RNA sequence in the present study was downloaded from the GEO database (https://www.ncbi.nlm.nih.gov/geo/) in asthma. The dataset of GSE76262 (118 asthma cases and 21 healthy controls) [10] was downloaded for differently expressed genes (DEGs) analysis under the cut-off criteria: |log2FC|> 1 and adjust *p* value < 0.05 and WGCNA analysis [11]. Then the overlapped genes of DEGs and the most significant module in the results of WGCNA analysis were selected by Venn package in R as candidate genes for subsequent GO/KEGG enrichment analysis and validation. The prediction of diagnostic capacity about the candidate gene was performed using the receiver operating characteristic (ROC) curve. The area under the curve (AUC) was used to evaluate the sensitivity and specificity of the selected gene. Finally, the most significantly expressed BIRC3 was obtained as our candidate gene. Then the correlation between BIRC3 expression and Th2 cytokines IL-4, IL-5, IL-13, and IL-25 in GSE76262 were analyzed. The BiocManager, org.Hs.eg.db, limma, WGCNA, Venn, enrichplot, ggplot2, and clusterProfiler packages in R language (version 4.0.3) were used in this study.

### Sputum collection of participants

Participants with stable asthma were recruited from the respiratory clinic and inpatient ward of the first affiliated hospital of Sun Yat-Sen University (Guangzhou, Guangdong, China). Asthma was diagnosed according to the following criteria: symptoms of wheezing, cough, and dyspnea; histamine provoking a 20% fall (PC20) of forced expiratory volume in the first second (FEV_1_) < 8 mg/ml and/or ≥ 12% increase in FEV_1_ following inhalation of 200 μg salbutamol. Healthy controls had no respiratory or inflammatory illness recruited by advertisement. Sputum induced by hypertonic saline (containing 4.5%NaCl) were collected under the operation of respiratory physicians. Then the sputum bolt was dissolved in 0.1% DL-Dithiothreitol (DTT, Sigma) [12]. The dissolved solution was filtered through a cell screen and centrifuged (1000 g 5 min, Eppendorf), the sputum cells in the sediment were collected in 1 ml TRIzol (Invitrogen, Carlsbad, USA) and the sputum supernatant were stored in − 80℃ refrigerator. Besides, other clinical information including the pulmonary function, peripheral blood total IgE, fractional exhaled nitric oxide (FeNO), and the percentage of eosinophils in peripheral blood (EOS%) of each participant were collected. All participants provided written informed consent. Studies were approved by the ethics committee of the first affiliated hospital of Sun Yat-Sen University.

### Cell culture and treatment

Human bronchial epithelial (HBEpiC) cells (ScienCell, #3210, USA) were cultured at the air–liquid interface as previously described [13] and stimulated with or without cytokines IL-4/IL-13 (10 ng/ml, Peprotech, USA) [14, 15]. Twenty-four hours after cytokines treatment, induced primary bronchial epithelial cells were collected using TRIzol reagent for subsequent RNA isolation and quantitative PCR detection.

### Quantitative real‐time polymerase chain reaction (qRT-PCR)

Total RNA from induced sputum cells and treated primary bronchial epithelial cells was isolated using TRIzol reagent according to the manufacturer’s instructions. Reverse transcribed using the Evo M-MLV RT Premix kit (AG, Hunan, China), the reaction conditions were 37 °C for 15 min, 85 °C for 5 s. The expression of candidate gene was quantitatively determined using Biosystems Light Cycler 480 (Applied Biosystems, Massachusetts, USA) as standard procedures. The primers used were as follows. BIRC3: forward, 5′-AAGCTACCTCTCAGCCTACTTT-3′, reverse, 5′-CCACTGTTTTCTGTACCCGGA-3′. GAPDH: forward, 5ʹ-ACCCAGAAGACTGTGGATGG-3ʹ, reverse, 5ʹ-TTCTAGACGGCAGGTCAGGT-3ʹ.

### Enzyme-linked immunosorbent assay (ELISA)

The protein levels of BIRC3, IL-4, IL-5, IL-13, IL-25, IL-10, IL-33, and TSLP in induced sputum supernatant were measured using commercially available ELISA kits (MEIMIAN, Jiangsu, China): BIRC3 (MM-50553H1), IL-4 (MM-0051H1), IL-5 (MM-0050H1), IL-13 (MM-0062H1), IL-25 (MM-1954H1), IL-10 (MM-0066H1), IL-33 (MM-1743H1), and TSLP (MM-0260H1) according to the manufacturer's instructions. All standards were measured in duplicate.

### Statistical analysis

R language (version 4.0.3) and GraphPad (version 8.3.0) software were used for all statistical analyses. For normally distributed data, we calculated means ± standard deviation (SD) and used Student’s t-test to compare across groups. For non-normally-distributed data, we calculated medians with interquartile ranges and used non-parametric tests (Mann–Whitney test). We analyzed correlation using Spearman's rank-order correlation. *p* value < 0.05 was considered statistical significance.

## Results

### Bioinformatic analysis of BIRC3

**Fig. 1:**
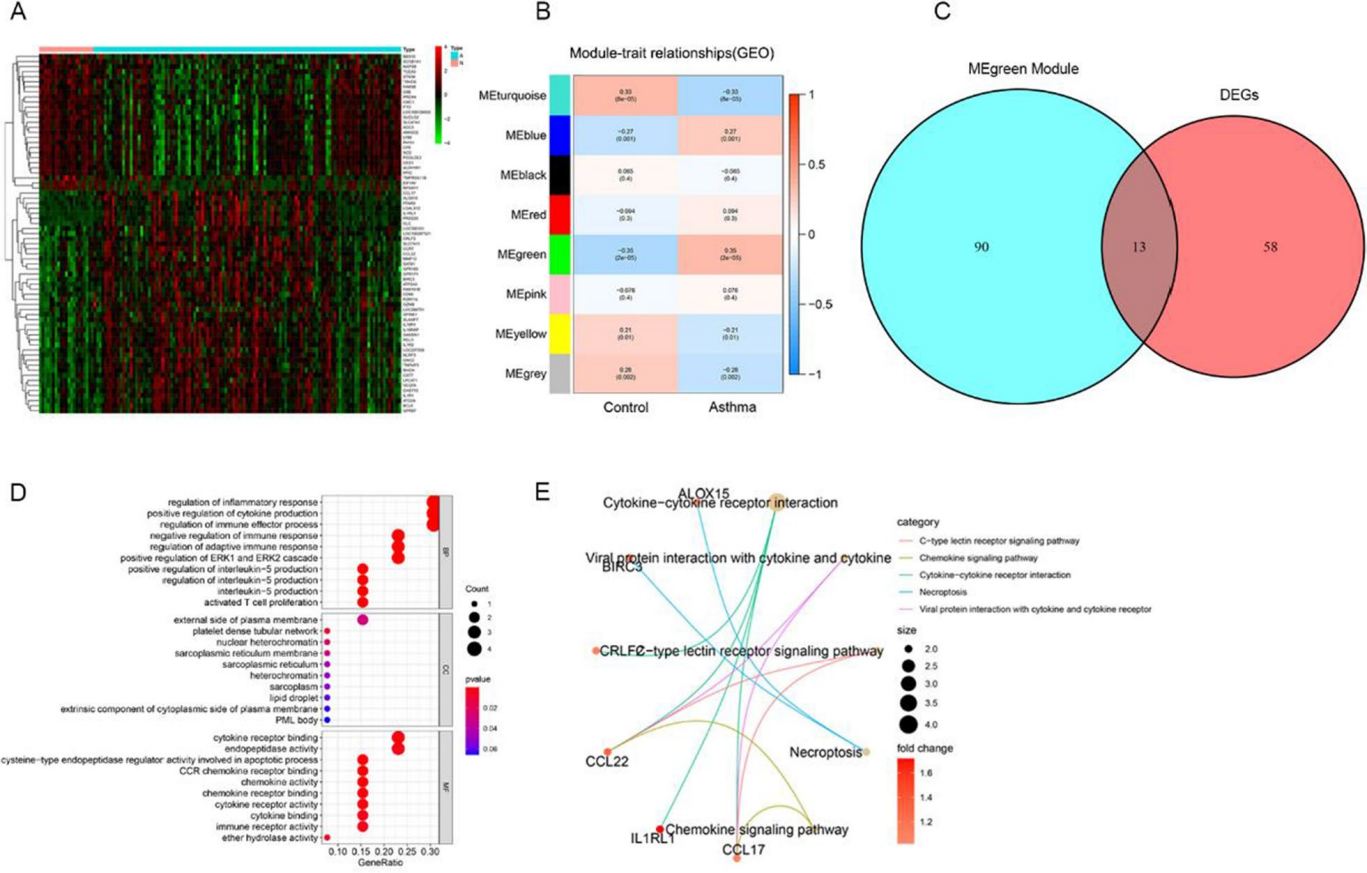
Screening overlapped genes in GSE7626. **A** Heatmap of DEGs between 118 asthma and 21 healthy controls in the GSE76262 dataset. Red and green, represent upregulated and downregulated, values; **B** Coexpression network of the GSE76262 dataset. The green module shows the most significant correlation with asthma; **C** Overlapped genes in DEGs and green module genes of WGCNA; GO (**D**) and KEGG (**E**) analysis of overlapped genes

**Fig. 2:**
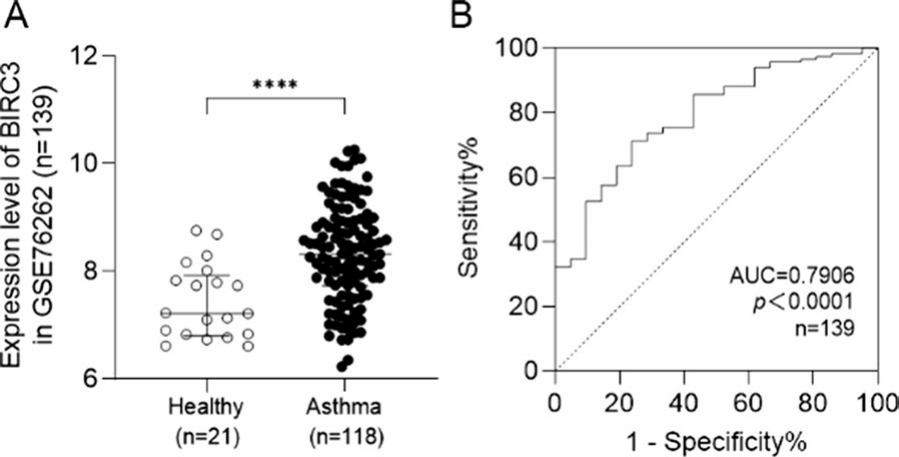
**A** The expression of BIRC3 was significantly upregulated in the GSE76262 dataset; **B** The ROC curve of BIRC3 expression in the GSE76262 dataset. For non-normally-distributed data, we calculated medians with interquartile ranges and used non-parametric tests (Mann–Whitney test). ****p < 0.0001

Based on the predetermined screened criteria (|log2FC|> 1 and adjust *p* value < 0.05), a total of 71 differentially expressed genes (DEGs) between asthma and healthy controls in GSE76262 were obtained, including 44 up-regulated and 27 down-regulated genes, respectively (Fig. 1A). Eight modules (Fig. 1B) were obtained by WGCNA, in which MEgreen showed a strong correlation to asthma (*r*_*s*_ = 0.69, *p* = 2.8e−17) (Additional file 1: Fig. S1). Then the overlapped genes between the green module and DEGs were filtered by the Venn package in R. A total of 13 genes, namely BIRC3, CLC, CCL17, IL1RL1, CCL22, ATP2A3, SATB1, CRLF2, LGALS12, PRSS33, ALOX15, FFAR3, and MMP12, were selected as overlapped genes (Fig. 1C, Additional file 1: Table S1). Through GO and KEGG enrichment analysis, we found these overlapped genes mainly contribute to the regulation of inflammatory response, cytokine production, immune effector process, cytokine receptor binding through cytokine-cytokine receptor interaction, and viral protein interaction with cytokine and cytokine receptor pathways (Fig. 1D, E). Finally, we selected the most significant differentially expressed gene BIRC3 (*p* = 2.85E−06) as the signature gene for further validation, which was significantly upregulated in asthma (*p* < 0.0001) (Fig. 2A). In the ROC analysis of the GSE76262 dataset, the area under the curve (AUC) of ROC was 0.7906 (*p* < 0.0001), meaning that BIRC3 expression has a good diagnostic capacity in asthma (Fig. 2B).

### Increased BIRC3 positively correlated with cytokines IL-4, IL-5, IL-13, and IL-25 in GSE76262 dataset

**Fig. 3:**
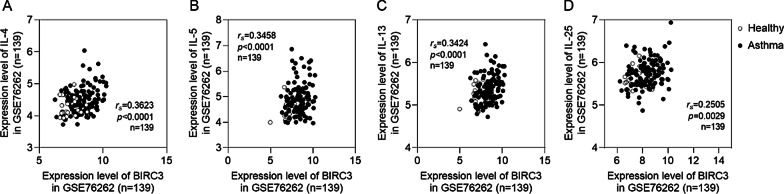
BIRC3 expression was correlated with cytokines IL-4 (**A**), IL-5 (**B**), IL-13 (**C**), and IL-25 (**D**) in the GSE76262. We analyzed correlation using Spearman’s rank-order correlation

Through GO and KEGG analysis, we found that the above 13 overlapped genes mainly contribute to the regulation of inflammatory response and cytokine production. Hence, we further explore the correlation between BIRC3 and inflammatory cytokines. Interestingly, the expression of BIRC3 in GSE76262 was significantly positively correlated with inflammatory cytokines IL-4, IL-5, IL-13, and IL-25 expression (*p* < 0.05) (Fig. 3A–D), which implied that BIRC3 may play an important role in the pathogenesis of inflammation in asthma.

### Both the mRNA and protein levels of BIRC3 were increased in induced sputum of asthma

**Fig. 4:**
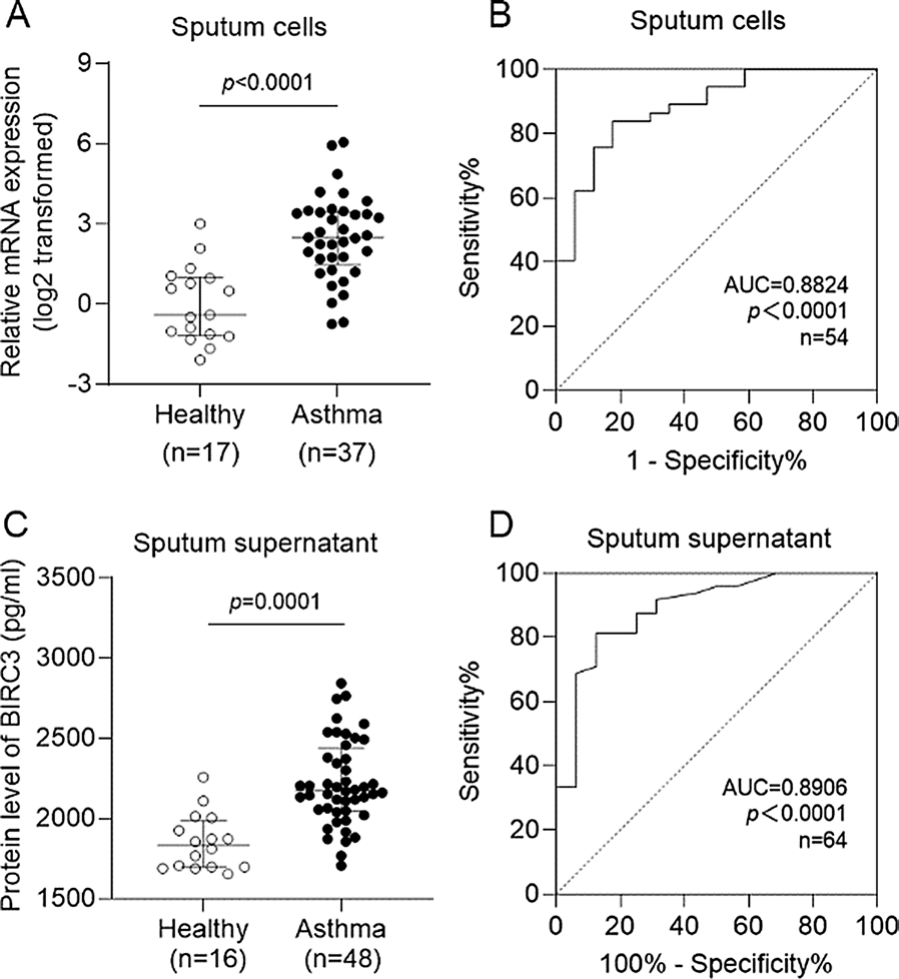
**A** BIRC3 mRNA expression in induced sputum cells (37 asthmatic patients vs 17 healthy controls) was detected by qRT-PCR; **B** The ROC curve of BIRC3 mRNA expression estimates the diagnostic capacity in induced sputum cells; **C** The protein level of BIRC3 was increased in induced sputum supernatant; **D** The ROC curve of BIRC3 protein level in induced sputum. For non-normally-distributed data, we calculated medians with interquartile ranges and used non-parametric tests (Mann–Whitney test)

To validate the clinical implication of BIRC3 in induced sputum of asthma, 21 healthy controls, and 55 asthmatic patients were recruited. There was no significant difference in age, sex, and BMI between the two groups (Table 1). BIRC3 mRNA was significantly upregulated in asthmatic patients (*p* < 0.0001) (Fig. 4A) through qRT-PCR and the AUC of the ROC curve was 0.8824 (*p* < 0.0001) (Fig. 4B). Consistently, BIRC3 protein in induced sputum supernatant remarkably increased in asthma (*p* = 0.0001) (Fig. 4C), and the AUC of the ROC curve was 0.8906 (*p* < 0.0001) (Fig. 4D), which further demonstrate a good diagnostic capacity of BIRC3 in asthma. These results are consistent with the GSE76262 microarray data.

**Table 1:** Characteristics of participants

	Healthy control (n = 21)	Asthma (n = 55)	*p* value
Age (years)	43.38 ± 15.95	36.90 ± 16.3	0.1199
Sex, M:F (%F)	10:11 (52.4%)	32:23 (41.8%)	0.4076
BMI (kg/m^2^)	22.29 ± 3.62	23.17 ± 3.06	0.3009
FEV_1_, % predicted	95.72 ± 20.94	82.80 ± 24.77	0.0413
EOS%	2.92 ± 3.93	4.90 ± 4.36	0.0782
IgE (IU/ml)	84.88 ± 64.56	219.05 ± 199.12	0.0059
FeNO (ppb)	16.78 ± 7.24	56.57 ± 49.83	0.0013

Values were presented as mean ± SD

*FeNO* fraction of exhaled nitric oxide, *FEV1* forced expiratory volume in the first second, t-tests for continuous variables and a chi-squared test for sex were used

### The mRNA and protein level of BIRC3 is associated with indicators of airway eosinophilic inflammation and peripheral blood allergic inflammation

**Fig. 5:**
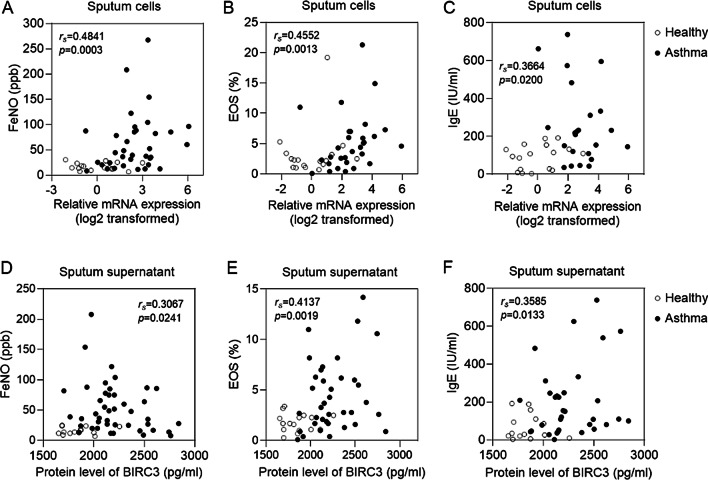
BIRC3 expression was correlated with asthmatic airway eosinophilic inflammation and peripheral blood allergic inflammation. BIRC3 mRNA (**A**–**C**) expression and protein level (**D**–**F**) were correlated with airway eosinophilic inflammation FeNO, peripheral blood EOS%, and serum total IgE. We analyzed correlation using Spearman's rank-order correlation

To further explore how BIRC3 affect eosinophilic inflammation, we analyzed the correlation between BIRC3 expression with clinical eosinophilic inflammation indicators. The results showed that BIRC3 mRNA in induced sputum cells was positively correlated with FeNO (r_s_ = 0.4841, *p* = 0.0003), EOS% (r_s_ = 0.4552, *p* = 0.0013) and IgE (r_s_ = 0.3664, *p* = 0.02) (Fig. 5A–C). Also, BIRC3 protein in induced sputum supernatant was positively correlated with FeNO (r_s_ = 0.3067, *p* = 0.0241), EOS% (r_s_ = 0.4137, *p* = 0.0019) and IgE (r_s_ = 0.3585, *p* = 0.0133) (Fig. 5D–F). These data suggested that BIRC3 might affect asthmatic eosinophilic and allergic inflammation.

**Fig. 6:**
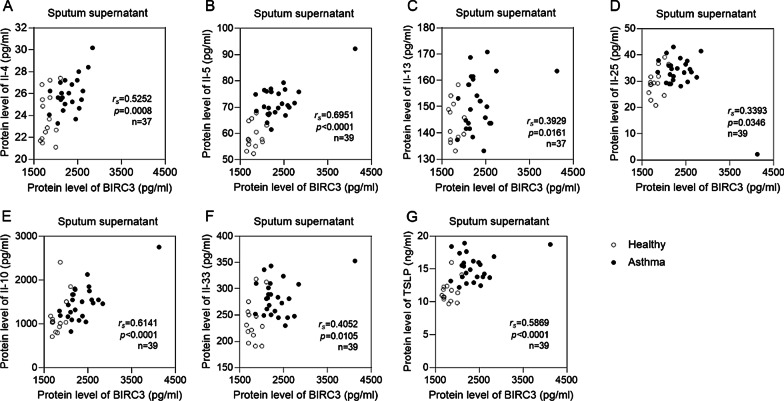
The protein levels of BIRC3 were correlated with cytokines IL-4 (**A**), IL-5 (**B**), IL-13 (**C**), IL-25 (**D**), IL-10 (**E**), IL-33 (**F**), and TSLP (**G**) in induced sputum. We analyzed correlation using Spearman's rank-order correlation

**Fig. 7:**
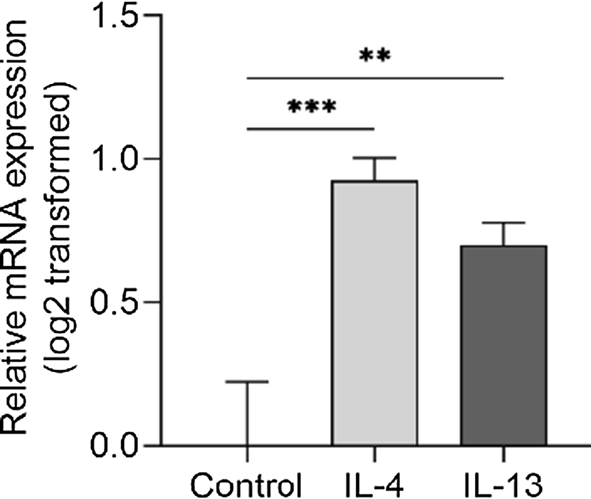
BIRC3 expression was upregulated in primary bronchial epithelial cells treated with cytokines IL-4/IL-13. For normally distributed data, we calculated means ± standard deviation (SD) and used parametric test one-way ANOVA to compare across groups. **p < 0.01, ***p < 0.001

Following by, we detected inflammatory cytokines in the induced sputum of asthma. Our results showed that BIRC3 expression was significantly correlated with IL-4 (r_s_ = 0.5252, *p* = 0.0008), IL-5 (r_s_ = 0.6951, *p* < 0.0001), IL-13 (r_s_ = 0.3929, *p* = 0.0161) and IL-25 (r_s_ = 0.3393, *p* = 0.0346) (Fig. 6A–D), which kept consistent with bioinformatics of GSE76262 dataset. Besides, we found a positive correlation between BIRC3 and IL-10, IL-33, and TSLP in induced sputum of asthma (*p* < 0.05, Fig. 6E–G). Furthermore, BIRC3 mRNA could be induced in primary bronchial epithelial cells treated with IL-4/IL-13 cytokines (*p* < 0.01, Fig. 7). Taken together, the above findings indicated that the increased BIRC3 may be involved in airway inflammation by affecting the secretion of Th2 cytokines.

### BIRC3 expression in induced sputum was negatively correlated with the pulmonary function

**Fig. 8:**
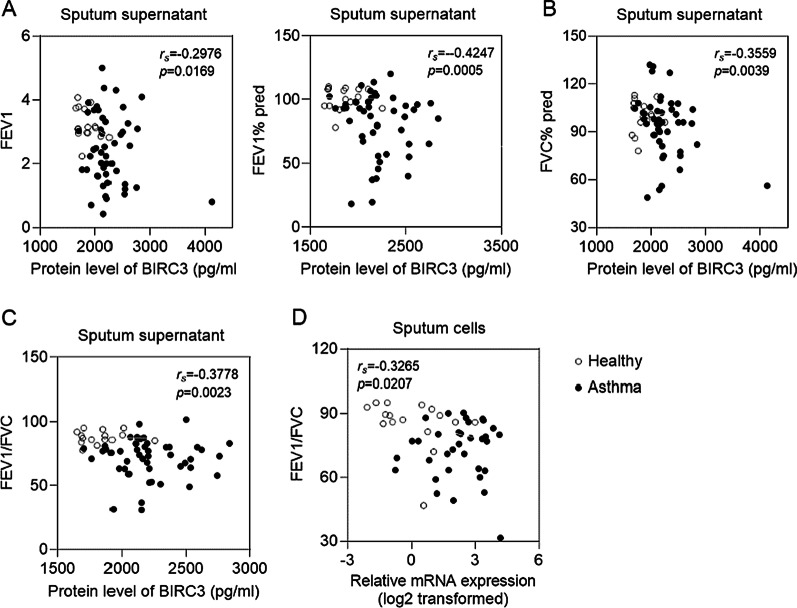
BIRC3 expression in induced sputum was negatively correlated with pulmonary function. **A**–**C** The protein level of BIRC3 was significantly negatively correlated with FEV_1_, FEV1% pred, FVC% pred, and FEV_1_/FVC; **D** The mRNA level of BIRC3 in induced sputum cells was significantly negatively correlated FEV_1_/FVC

To further explore the function of BIRC3 in pulmonary function, we analyzed the correlation between BIRC3 and pulmonary function. Our results showed that BIRC3 protein in induced sputum supernatant was negatively correlated with FEV_1_, FEV_1_% pred, FVC% pred, and FEV_1_/FVC (Fig. 8A–C), and BIRC3 mRNA in induced sputum cells was negatively correlated with FEV_1_/FVC (Fig. 8D), indicating that the highly expressed BIRC3 had worse lung function.

## Discussion

Asthma is characterized by chronic airway inflammation [16, 17], in which Th2 cells promote the accumulation of eosinophils by secreting interleukin IL-4, IL-5, and IL-13 is the key pathogenesis of asthma [18–21]. Biomarkers in induced sputum provide a noninvasive method of assessing and monitoring the pathogenesis of airway diseases [4, 6]. In the present study, we identified BIRC3, a sputum biomarker, and explored its potential role in asthma using bioinformatical analysis. Next, we verified its expression in clinical induced sputum samples. Finally, we found that increased BIRC3 was positively correlated with airway eosinophilic inflammation, Th2 cytokines secretion, and airway obstruction, indicating that upregulated BIRC3 may participate in the pathogenesis of asthma.

It was reported that BIRC3 was upregulated in asthma [22, 23] and was predicted as a pathogenic gene involved in childhood asthma [24]. Similarly, we found that BIRC3 mRNA and protein were significantly upregulated in asthma, and the results of AUC demonstrated its good diagnostic capacity. Besides, we found that patients with highly expressed BIRC3 exhibited worse pulmonary function. To some extent, our study validated the effect of BIRC3, a pathogenic gene, in asthma.

As our results show, the 13 overlapped genes were significantly involved in the immune and inflammatory response. It is well known that Th2 cytokines are critical in the pathogenesis of allergic disorders [25, 26]. Accordingly, we explored the interaction between BIRC3 expression and Th2 cytokines in GSE76262. And we found a positive correlation between BIRC3 and IL-4, IL-5, IL-13, and IL-25. Consistently, we detected IL-4, IL-5, IL-13, IL-25, IL-10, IL-33, and TSLP in induced sputum of asthma and proved a positive correlation between BIRC3 and these cytokines. Furthermore, the expression of BIRC3 could be induced in primary bronchial epithelial cells stimulated with IL-4/IL-13 cytokine. Beyond that, we found that BIRC3 mRNA and protein are correlated with eosinophilic and allergic inflammation indicators, including FeNO, eosinophils, and IgE. Therefore, we speculated that upregulated BIRC3 potentially affects Th2 immune inflammation to participate in airway eosinophilic and allergic inflammation of asthma.

To search the underlying mechanism regulated by BIRC3 in asthma, we conducted KEGG pathway enrichment using 13 overlapped genes and found that BIRC3 is mainly enriched in cell apoptosis and necrosis. Nemours studies found that BIRC3 protein inhibits apoptosis [22, 27], which could impact susceptibility to asthma [28–30]. However, there are few reports about the mechanism of BIRC3 involved in asthma, subsequent experiments' validations are needed.

Overall, induced sputum BIRC3 overexpression could predict heavier airway inflammation and airway obstruction. Upregulated BIRC3 may act as a pathogenic gene involved in asthma through Th2 cytokines activation. However, more experiments’ verifications are needed to explore the exact molecular mechanism of BIRC3 in asthma.

## Conclusions

In conclusion, we identified BIRC3 mRNA and protein significantly increased in induced sputum of asthma and were positively correlated with Type 2 airway inflammation. BIRC3 exhibits a noninvasive diagnostic value and targeted inhibition of BIRC3 might be a potential asthma therapy.

## Supplementary Information

**Additional file 1.** The correlation of green module with asthma in WGCNA analysis and the expression of 13 intersected genes in GSE76262.

## Data Availability

We declared that the data described in the manuscript are freely available to any scientist wishing to use them for non-commercial purposes, without breaching participant confidentiality.

## Abbreviations

BIRC3: Baculoviral IAP repeat-containing 3
qRT-PCR: Quantitative real‐time polymerase chain reaction
ELISA: Enzyme-linked immunosorbent assay
FeNO: Factional exhaled nitric oxide
IgE: Immunoglobulin E
EOS%: The percentage of eosinophils in peripheral blood
FEV1: Forced expiratory volume in the first second
FVC: Forced vital capacity
FEV1/FVC: Forced vital capacity rate of one second
GINA: Global initiative for asthma
GEO: Gene expression omnibus
DTT: DL-dithiothreitol
HBEpiC: Human bronchial epithelial cells
ROC: Receiver operating characteristic curve
AUC: The area under ROC curve

## References

[1] Mims J (2015). Asthma: definitions and pathophysiology. Int Forum Allergy Rhinol.

[2] Boulet L, Reddel H, Bateman E, Pedersen S, FitzGerald J, O'Byrne P (2019). The Global Initiative for Asthma (GINA): 25 years later. Eur. Respir J.

[3] Baines KJ, Simpson JL, Wood LG, Scott RJ, Gibson PG (2011). Transcriptional phenotypes of asthma defined by gene expression profiling of induced sputum samples. J Allergy Clin Immunol.

[4] Winter NA, Qin L, Gibson PG, McDonald VM, Baines KJ, Faulkner J (2021). Sputum mast cell/basophil gene expression relates to inflammatory and clinical features of severe asthma. J Allergy Clin Immunol.

[5] Fricker M, Gibson PG, Powell H, Simpson JL, Yang IA, Upham JW (2019). A sputum 6-gene signature predicts future exacerbations of poorly controlled asthma. J Allergy Clin Immunol.

[6] Chung K, Adcock I (2019). Precision medicine for the discovery of treatable mechanisms in severe asthma. Allergy.

[7] Baines K, Fricker M, McDonald V, Simpson J, Wood L, Wark P (2020). Sputum transcriptomics implicates increased p38 signalling activity in severe asthma. Respirology (Carlton, Vic.).

[8] Li Q, Baines K, Gibson P, Wood L (2016). Changes in expression of genes regulating airway inflammation following a high-fat mixed meal in asthmatics. Nutrients.

[9] Baines K, Simpson J, Wood L, Scott R, Fibbens N, Powell H (2014). Sputum gene expression signature of 6 biomarkers discriminates asthma inflammatory phenotypes. J Allergy Clin Immunol.

[10] Kuo C, Pavlidis S, Loza M, Baribaud F, Rowe A, Pandis I (2017). T-helper cell type 2 (Th2) and non-Th2 molecular phenotypes of asthma using sputum transcriptomics in U-BIOPRED. Eur Respir J.

[11] Abu-Jamous B, Kelly S (2018). Clust: automatic extraction of optimal co-expressed gene clusters from gene expression data. Genome Biol.

[12] Guiot J, Demarche S, Henket M, Paulus V, Graff S, Schleich F (2017). Methodology for sputum induction and laboratory processing. J Vis Exp.

[13] Tanabe T, Rubin B (2016). Airway goblet cells secrete pro-inflammatory cytokines, chemokines, and growth factors. Chest.

[14] Kikuchi T, Shively J, Foley J, Drazen J, Tschumperlin D (2004). Differentiation-dependent responsiveness of bronchial epithelial cells to IL-4/13 stimulation. Am J Physiol Lung Cell Mol Physiol.

[15] Gour N, Wills-Karp M (2015). IL-4 and IL-13 signaling in allergic airway disease. Cytokine.

[16] Papi A, Brightling C, Pedersen S, Reddel H (2018). Asthma. Lancet (London, England).

[17] Lambrecht B, Hammad H, Fahy J (2019). The cytokines of asthma. Immunity.

[18] Agache I, Akdis C (2019). Precision medicine and phenotypes, endotypes, genotypes, regiotypes, and theratypes of allergic diseases. J Clin Investig.

[19] Agache I, Sugita K, Morita H, Akdis M, Akdis C (2015). The complex type 2 endotype in allergy and asthma: from laboratory to bedside. Curr Allergy Asthma Rep.

[20] Bonser L, Erle D (2019). The airway epithelium in asthma. Adv Immunol.

[21] Huang C, Li F, Wang J, Tian Z (2020). Innate-like lymphocytes and innate lymphoid cells in asthma. Clin Rev Allergy Immunol.

[22] Bazan-Socha S, Buregwa-Czuma S, Jakiela B, Zareba L, Zawlik I, Myszka A (2021). Reticular basement membrane thickness is associated with growth- and fibrosis-promoting airway transcriptome profile-study in asthma patients. Int J Mol Sci.

[23] Roscioli E, Hamon R, Ruffin RE, Grant J, Hodge S, Zalewski P (2016). BIRC3 single nucleotide polymorphism associate with asthma susceptibility and the abundance of eosinophils and neutrophils. J Asthma.

[24] Gao XM (2016). A network approach predicts NFKBIA and BIRC3 as pathogenic genes in childhood asthma. Genet Mol Res.

[25] Harb H, Chatila T (2020). Mechanisms of dupilumab. Clin Exp Allergy J Br Soc Allergy Clin Immunol.

[26] Zissler U, Esser-von Bieren J, Jakwerth C, Chaker A, Schmidt-Weber C (2016). Current and future biomarkers in allergic asthma. Allergy.

[27] Rada M, Nallanthighal S, Cha J, Ryan K, Sage J, Eldred C (2018). Inhibitor of apoptosis proteins (IAPs) mediate collagen type XI alpha 1-driven cisplatin resistance in ovarian cancer. Oncogene.

[28] Paivandy A, Pejler G (2021). Novel strategies to target mast cells in disease. J Innate Immun.

[29] Lambrecht B, Hammad H (2013). Death at the airway epithelium in asthma. Cell Res.

[30] Sauler M, Bazan I, Lee P (2019). Cell death in the lung: the apoptosis-necroptosis axis. Annu Rev Physiol.

