# Supplementary material for: Exploration of induced sputum BIRC3 levels and clinical implications in asthma
Source: BMC Pulm Med. 2022 Mar 14;22:86. doi: 10.1186/s12890-022-01887-2 (PMC8922789; doi:10.1186/s12890-022-01887-2)
Supplement: Supplementary file 1 — Additional file 1. The correlation of green module with asthma in WGCNA analysis and the expression of 13 intersected genes in GSE76262. [file 12890_2022_1887_MOESM1_ESM.docx]

**Additional file 1**


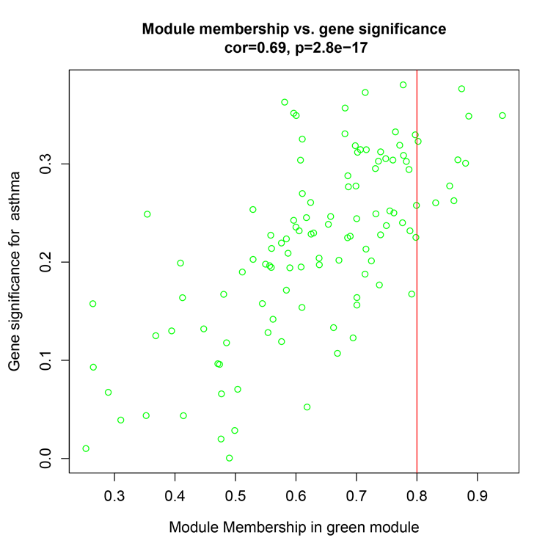


**Sup.Fig.1** The green module showed the strongest correlation with asthma among the eight modules.

| **Sup. Table 1** The expression of 13 intersected genes in the GSE76262 dataset. | | | | | |
| --- | --- | --- | --- | --- | --- |
| Gene | logFC | AveExpr | p.Value | adj.p.Val |  |
| BIRC3 | 1.106199379 | 8.185755654 | 2.85E-06 | 0.001889021 |  |
| CLC | 2.219468227 | 5.591699318 | 3.56E-06 | 0.002027461 |  |
| CCL17 | 1.063777692 | 5.69414602 | 8.64E-06 | 0.002774718 |  |
| IL1RL1 | 1.717433038 | 5.99211947 | 1.91E-05 | 0.003799064 |  |
| CCL22 | 1.277596653 | 7.592019813 | 1.93E-05 | 0.003804692 |  |
| ATP2A3 | 1.004817917 | 6.509452539 | 2.03E-05 | 0.003853814 |  |
| SATB1 | 1.03451805 | 8.468448443 | 0.000104603 | 0.006831421 |  |
| CRLF2 | 1.076970371 | 6.103713601 | 0.000131614 | 0.007598898 |  |
| LGALS12 | 1.088328733 | 5.448706212 | 0.000223585 | 0.009556008 |  |
| PRSS33 | 1.204815045 | 6.110207977 | 0.000264902 | 0.010186436 |  |
| ALOX15 | 1.02188794 | 5.114945971 | 0.000347309 | 0.01136709 |  |
| FFAR3 | 1.072891219 | 5.196910935 | 0.001541423 | 0.023155063 |  |
| MMP12 | 1.216084792 | 7.211174991 | 0.005323883 | 0.042760652 |  |
| logFC: fold change; AveExpr: Average expression; adj.P.Val: adjust.p.value. | | | | |  |
